# Rapid Assessment of Tumor Thickness in Cutaneous Squamous Cell Carcinoma Using Ex Vivo Confocal Microscopy

**DOI:** 10.3390/cancers18020228

**Published:** 2026-01-12

**Authors:** Daniela Hartmann, Katharina Wex, Aimée Braun, Paulina Pabst, Alisa Swarlik, Lisa Buttgereit, Lara Stärr, Andreas Ohlmann, Elke C. Sattler, Maximilian Deußing

**Affiliations:** 1 Department of Dermatology and Allergy, LMU University Hospital, Ludwig Maximilian University of Munich, 80337 Munich, Germany; 2 Department of Dermatology and Allergy, Munich Municipal Hospital, 80337 Munich, Germany; 3 Department of Ophthalmology, LMU University Hospital, Ludwig Maximilian University of Munich, 80337 Munich, Germany

**Keywords:** non-melanocytic skin cancer, cutaneous squamous cell carcinoma, tumor thickness, high-risk tumor, dermatosurgery, digital pathology, ex vivo confocal microscopy, histopathological images

## Abstract

**Objectives**: Ex vivo confocal laser scanning microscopy (EVCM) is a pioneering diagnostic method that enables fresh tissue samples to be analyzed directly during surgery. For the assessment of non-melanocytic skin cancer (NMSC), including cutaneous squamous cell carcinoma (cSCC), it provides a rapid addition to conventional histology. While previous studies have shown that EVCM reliably identifies the morphological features of cSCCs, quantitative criteria such as tumor thickness have not yet been systematically evaluated. This study investigated whether EVCM can be used to accurately and reproducibly measure the thickness of cSCCs, an important parameter for predicting metastatic risk. **Methods**: Eighty-two histologically verified cSCCs from different anatomical sites were assessed by the current gold standard of histopathology and EVCM. A statistical comparison of the confocal tumor thickness (CTT) and the histopathological tumor thickness (HTT) was then performed. In addition, it was analyzed how reliable EVCM was in the assignment of cSCCs to the correct tumor thickness category. **Results**: There was a very high agreement between both methods, evidenced by a Spearman correlation coefficient of 0.94 and a coefficient of determination of 0.859. Overall, 95.1% of the samples were correctly classified into the appropriate tumor thickness category using EVCM. Cohen’s Kappa of 0.90 indicated almost perfect agreement between EVCM and histology. **Conclusions**: These findings demonstrate that EVCM is a precise and reliable method for determining tumor thickness and the corresponding category in cSCCs. It enables immediate intraoperative assessment of the metastatic risk and preliminary classification of low-risk tumors. Additional studies with larger patient cohorts are required to further validate these results and support clinical implementation.

## Simple Summary

In recent decades, the incidence of non-melanocytic skin cancer (NMSC) has steadily increased in Germany and other industrial countries, creating a demand for rapid and reliable diagnostic methods. Ex vivo confocal laser scanning microscopy (EVCM) represents a promising approach, as it allows for intraoperative analysis of fresh tissue within minutes. Recent studies have shown that EVCM can reliably detect morphological features of cutaneous squamous cell carcinoma (cSCC). The aim of this study was to evaluate how accurately EVCM can determine tumor thickness in cSCCs and assign them to the correct tumor thickness categories, a key parameter for predicting metastatic risk. The results demonstrate that EVCM provides precise and reproducible measurements, indicating its potential to enable intraoperative assessment of metastatic risk and early identification of low-risk tumors in clinical practice.

## 1. Introduction

Non-melanocytic skin cancer (NMSC) is the most common type of cancer with around 1.2 million new cases in 2020 worldwide [1], with cutaneous squamous cell carcinoma (cSCC) being the second most frequent form of NMSC following basal cell carcinoma (BCC). The incidence of NMSC has been continuously increasing in recent decades, both in Germany and other industrial countries [2,3,4,5]. For Germany, according to the German Federal Statistical Office, a total of 91,000 inpatient treatment cases for NMSC were recorded in 2023, which corresponds to an increase of 117% within 20 years (2003: 41,900 inpatient treatment cases) [6]. At the same time, there will be a growing shortage of dermatologists in Germany in the coming years, which will lead to an undersupply, particularly in rural areas [7]. Given these developments, there is an urgent need for efficient and reliable diagnostic methods. In this context, ex vivo confocal laser scanning microscopy (EVCM) is an innovative diagnostic technique that enables precise and rapid tissue assessment. Compared to the gold standard of histopathology, this method eliminates the time-consuming process of preparing paraffin blocks and sections. In contrast, EVCM provides digitally simulated Hematoxylin and Eosin (H&E) images within a few minutes, which are nearly similar to those of conventional histopathology [8]. This means that EVCM can be used intraoperatively to provide dermatologists with direct histopathological feedback on the fresh tissue removed [9]. Particularly in the surgical management of cSCC, in addition to confirming the underlying pathology, the exact measurement of tumor thickness is of crucial prognostic importance, as it directly correlates to the metastatic risk and therefore influences further clinical procedure and follow-up decisions [10]. While classical histopathology remains the gold standard for tumor thickness assessment, the rapid diagnostic capability of the EVCM method, as already demonstrated in other diagnostic applications such as surgical margin monitoring, supports faster clinical decision-making and contributes to simplified patient management [11]. In addition, EVCM, together with the establishment of telemedicine applications, could help to close healthcare gaps in the future.

Several studies have already been conducted on EVCM, demonstrating its potential in the diagnosis of various tumor entities. For example, a specificity of 97% and a sensitivity of 93% have been achieved for the diagnosis of SCC compared to conventional histopathology [12]. In addition, its use for measuring tumor thickness has already been investigated in malignant melanomas, where EVCM proved to be a reliable and precise method in comparison with the standard histopathological approach [13]. Nevertheless, it remains unclear whether EVCM can also provide reliable results for tumor thickness determination in cSCCs. Therefore, the aim of this study was to investigate the extent to which EVCM provides consistent results for tumor thickness measurement in this tumor entity, and how well the method can assign the cSCC to the corresponding tumor thickness category, which is particularly important for the assessment of the risk of metastasis.

## 2. Materials and Methods

### 2.1. Study Participants

From September 2020 to February 2025, patients with suspected cSCCs or other conspicuous skin lesions indicative of NMSC were enrolled at the Department of Dermatology and Allergology at Ludwig Maximilian University (LMU) in Munich. Adult patients aged 18 and over with suspicious skin abnormalities, either presented for the first time or for follow-up, were included. Immunosuppressed patients were also enrolled if all inclusion criteria were fulfilled. Recruitment took place via the outpatient clinic, as well as through both the ambulatory and inpatient surgical units. All participants were comprehensively informed about the investigation and gave their written consent to participate. The study project and the informed consent forms were previously reviewed and approved by the local ethic committee of the LMU University Hospital in Munich (Ref.-Nr. 19-150 and Ref.-Nr. 23-0393). For the final analysis, only cases with histologically confirmed cSCCs were selected, which resulted in 82 cases.

### 2.2. Study Design and Ex Vivo Confocal Laser Scanning Microscopy

**Figure 1:**
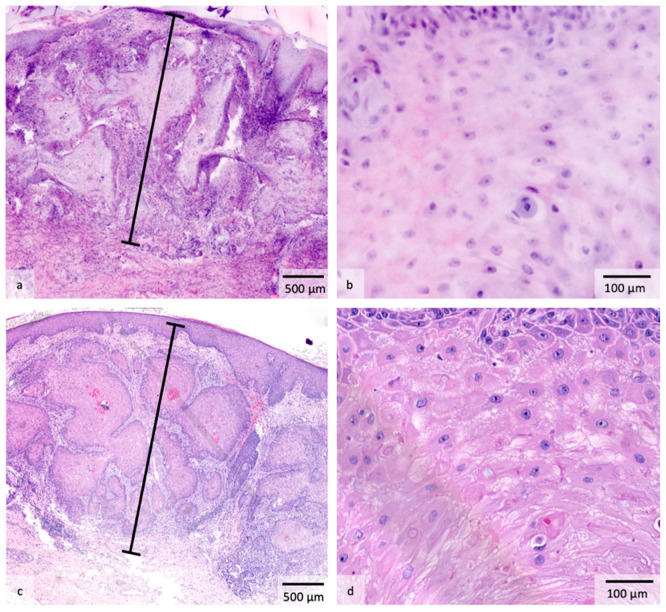
H&E-stained images of a cutaneous squamous cell carcinoma with measured tumor thickness of approximately 3 mm. (**a**) Overview image acquired using EVCM; (**b**) Detailed EVCM image showing atypical tumor cell clusters of keratinocytes; (**c**) Overview of the corresponding conventional histopathological section; (**d**) Corresponding detailed image of the histopathological section highlighting atypical tumor cell clusters of keratinocytes.

The study was structured as follows. A biopsy was first taken from each suspicious skin lesion. Afterwards, the fresh tissue was placed in saline solution to ensure safe transport. The samples were then stained according to a standardized protocol. The specimen was successively immersed for 30 s in ethanol (0.7 mmol/L), Acridine Orange (AO, 0.04 mmol/L, Sigma-Aldrich, St. Louis, MO, USA), FCF Fast Green (0.067 mmol/L, Sigma-Aldrich, St. Louis, MO, USA), and NaCl (0.09 mmol/L) [14,15]. Subsequently, the sample was placed cut-side down on a microscope slide and attached using sponges and magnets to stabilize the specimen during analysis. In the next step, imaging was performed using the VivaScope 2500M-G4, which was available at the Department of Dermatology and Allergology at the LMU Munich. Two different laser sources were used: a laser in the blue spectral range (488 nm) was applied to excite the fluorescent dye AO [16]. AO is a well-established coloring agent in histology that enables the targeted visualization of cellular structures [17,18]. In addition, a red laser with a wavelength of 638 nm was utilized to detect reflection signals from the tissue [16]. Both signal types (fluorescence and reflection) were recorded simultaneously and processed digitally. This was followed by an algorithm-supported conversion of the image data into pseudocolors, which were modeled on classic Hematoxylin and Eosin (H&E) staining. The process enables rapid and digitally supported assessment of the tissue samples in a display form that is comparable to conventional histopathological processing [17]. After EVCM imaging, the tumor thickness of the cSCCs was determined on the digital H&E images by an experienced EVCM examiner who is also a dermatopathologist (D.H.). The confocal tumor thickness (CTT) measurements were taken vertically from the stratum granulosum to the deepest infiltrating tumor cell and expressed in millimeters (mm) following standard histopathology practice [19]. The fresh tissue was then fixed in formalin and subjected to the regular histopathological procedure. Staining and EVCM imaging do not cause any permanent changes or damage to the corresponding specimen [12]. In this context, it is important to note that EVCM and conventional histopathology were performed on the same samples, including the initial biopsies, to ensure that the EVCM measurements were directly comparable with the corresponding histopathological findings. After preparing the respective paraffin blocks and sections, the histopathological tumor thickness (HTT) of the cSCCs was measured by another expert dermatopathologist in a blinded manner and according to the same measuring principle (Figure 1).

### 2.3. Statistical Analysis

CTT and HTT measurements were compared with each other using various statistical methods. The data analysis was performed with GraphPad Prism, version 10.4 (GraphPad Software, San Diego, CA, USA), beginning with descriptive statistics. The Wilcoxon sign-rank test was then applied to check whether there was a significant difference between the two methods. Agreement between measurements was assessed using a Bland–Altman plot and the strength of the association between HTT and CTT was evaluated with Spearman’s correlation coefficient. In addition, a linear regression was performed to analyze the existence of a linear relationship between the two measurements. To determine the correct group allocation, which is of clinical importance, the tumor thicknesses were categorized into three predefined categories (0–2 mm, 2.1–6 mm, >6 mm). This classification aligns with the metastasis risks outlined in the German S3 guideline “Actinic keratosis and cutaneous squamous cell carcinoma” [20]. In order to analyze the agreement of the categorization, the kappa test and Fisher’s exact test were utilized. Finally, the median and interquartile range (IQR) of measurement differences between EVCM and histopathology were calculated separately for the histopathological differentiation grades G1 and G2–3 to assess whether the degree of differentiation influenced EVCM measurements of tumor thickness. All statistical tests were selected accordingly due to the lack of normal distribution of the data. The significance level was set at *p* < 0.05 for all analyses.

## 3. Results

A total of 82 patients with 82 histologically confirmed cSCCs were included in the study. The patient population consisted of 19 women and 63 men, with a mean age of 78.7 years. Further demographic data as well as the descriptive statistics are shown in Table 1.

**Figure 2:**
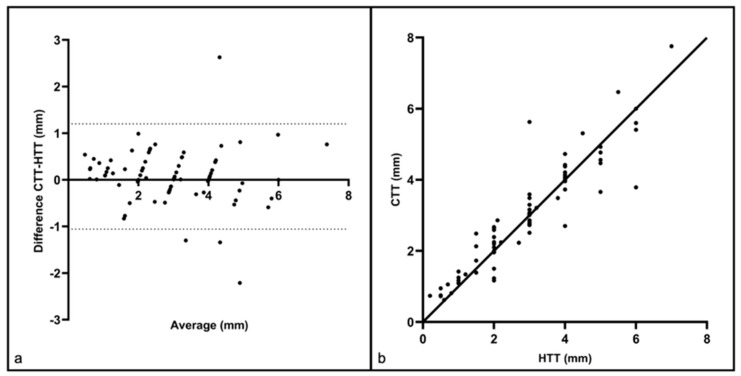
(**a**) Bland–Altman plot showing the agreement between the confocal tumor thickness (CTT) and the histopathological tumor thickness (HTT): Each point represents one sample. The mean difference (bias) was 0.072 mm (solid line), and the limits of agreement were −1.057 mm and 1.201 mm (dashed lines). (**b**) The correlation curve between the confocal tumor thickness (CTT) and the histopathological tumor thickness (HTT) demonstrated a strong correlation between the two variables with a Spearman correlation coefficient of r = 0.94. Each point in the diagram represents one patient sample. The solid line illustrates a perfect correlation (r = 1).

The Wilcoxon signed-rank test resulted in a *p*-value of 0.0634 and was therefore not statistically significant. Furthermore, a Bland-Altman plot was created to assess the agreement between the CTT and HTT measurements (Figure 2a). The results revealed a bias (mean difference between CTT and HTT) of 0.072 mm +/− SD 0.58 mm and limits of agreement (95% confidence interval of the differences) ranging from −1.057 mm to 1.201 mm. Although these absolute limits of agreements may appear substantial, particularly for tumors of small thickness, they do not adequately reflect the relative agreement between the methods across the entire spectrum of tumor sizes. Therefore, to better assess the clinical relevance, a relative Bland–Altman analysis was performed, which evaluates the differences in relation to the measured sizes of the tumor thicknesses. It revealed a relative bias of 6.0% with relative limits of agreement extending from −41% to 53%.

In addition, the Spearman correlation coefficient of r = 0.94 demonstrated that there is a strong and statistically significant positive correlation between HTT and CTT (*p* < 0.0001, Figure 2b). Furthermore, the performance of a linear regression showed that a strong and statistically significant linear relationship exists between HTT and CTT (*p* < 0.0001). The equation of the regression line was: CTT = 0.9033 × HTT + 0.3519. The coefficient of determination (R^2^) was 0.859.

Further analyses were carried out to assess how reliably the EVCM measurement method could assign the tumor thicknesses to their respective categories. The result of histopathological investigation served as the gold standard for this classification and the terms “correct” and “misclassified” refer to agreement or disagreement with the histopathological categorization. A total of 95.1% of all samples were correctly classified with the help of the EVCM measurement method (0–2 mm: 91.2% correct; 8.8% misclassified; 2.1–6 mm: 97.9% correct; 2.1% misclassified; >6 mm: 100% correct, based on a single sample, which limits the interpretability of this percentage). Furthermore, Cohen’s Kappa of 0.90 demonstrated that there was almost perfect agreement between the two measurement methods in the categorization of tumor thickness. In addition, Fisher’s Exact Test was performed to check whether the accuracy of tumor categorization differed between the various tumor thickness groups. The analysis revealed only non-significant *p*-values (*p*1 = 0.3041, *p*2 and *p*3 > 0.9999). For differentiation grade G1, the median measurement difference between EVCM and histopathology was 0.05 mm (IQR −0.11 to 0.30 mm), and for G2–3 it was 0.01 mm (IQR −0.04 to 0.39 mm).

## 4. Discussion

Our working group has previously explored the accuracy of tumor thickness measurement in malignant melanoma, providing an important basis for the present investigation [13]. In addition, further evaluations showed that the agreement was constant across all tumor thickness areas [15]. With the present study, this approach was transferred to cSCCs for the first time and tested on a larger number of cases (n = 82). Our results not only confirm the high agreement between CTT and HTT with a Spearman correlation coefficient of r = 0.94, but also show that the determination of tumor thickness using EVCM provides reliable and precise results for cSCCs in most tumor thickness categories (Cohen’s Kappa = 0.90; only non-significant Fisher’s exact tests). However, the measurement in the tumor thickness category of >6 mm was based on a single sample, representing a limitation of the study. Only four samples deviated from the limits of agreement in the absolute Bland–Altman analysis, with a maximum discrepancy of 2.63 mm (HTT: 3 mm, CTT: 5.63 mm, both remaining within the same clinically relevant thickness category). For five samples, the measured values matched exactly. This extends the validity of the EVCM measurement method to another important tumor entity.

Several studies have additionally demonstrated that cSCCs can be reliably identified on the basis of various morphological criteria in EVCM. In a previous study, we found that hyperkeratosis, erosions, bright and speckled cells, as well as cell nests in the dermis, keratin pearls, and peritumoral infiltration of inflammatory cells are common features that support the diagnosis of cSCCs in EVCM [21]. Negrutiu M. et al. were able to confirm these findings in a detailed study [22]. This reinforces that cSCCs can be clearly differentiated from other tumor entities using EVCM. Our study extends this approach by showing that, in addition to qualitative features, quantitative measurements of tumor thickness are possible. The descriptive statistics reveal that both measurement methods provide similar mean values (HTT: 2.890 mm and CTT: 2.963 mm), indicating comparable accuracy between EVCM and conventional histopathology. This observation was further supported by the Wilcoxon signed-rank test (*p* = 0.0634), showing no statistically significant difference between the methods. In addition, the Bland–Altman analysis revealed an absolute deviation of 0.072 mm, meaning that the EVCM measurements differed on average by less than one tenth of a millimeter from conventional histopathology. The high value of the coefficient of determination (R^2^ = 0.859) confirms that CTT enables a dependable estimation of HTT over the whole spectrum of measured values. Finally, the histopathological differentiation grades G1 and G2–3 showed comparable median values (G1: 0.05 mm; G2–3: 0.01 mm) for the measurement differences. This suggests that the grade did not influence the EVCM tumor thickness measurement. Moreover, our cohort represented a typical spectrum of degrees of differentiation in tertiary dermatological surgery centers.

Various studies have already shown that the risk of distant metastases in cSCCs rises with increasing tumor thickness [19,23,24,25,26,27]. This correlation was confirmed by a systematic review and meta-analysis by Thompson A. et al., who investigated risk factors for metastases in cSCCs in 36 studies. They found that the greatest risk ratio for metastases in cSCCs was tumor thickness [28]. This underlines the importance of a reliable and accurate measurement of tumor thickness. Additionally, our results show clearly that EVCM is able to classify tumor thickness with high accuracy into the corresponding category. The correct classification plays a decisive role in prognostic evaluation. In a prospective study of 615 patients, Brantsch K. et al. demonstrated metastasis rates of 0% (<2 mm), 4% (2.1–6 mm) and 16% (>6 mm) [10]. These results have been incorporated into the current German guidelines on “Actinic keratosis and cutaneous squamous cell carcinoma” in section “3.4 Prognostic factors for metastasis in invasive squamous cell carcinoma” [20], showing the importance of the factor tumor thickness in the prognostic classification of cSCCs. In our study, 95.1% of all samples were correctly classified by EVCM. Our investigation thus makes an important contribution to establishing EVCM as an efficient, rapid method for determining the tumor thickness of cSCCs. In contrast to conventional histopathology, which is associated with time lags due to the preparation of paraffin blocks and sections, EVCM enables immediate assessment through the digital representation of H&E-equivalent images in real-time. This opens up the possibility of an instant analysis of the individual risk of metastasis during the surgical procedure. Despite having a high overall agreement, the relative Bland–Altman limits of agreement (−41% to 53%) showed some variability, which is especially critical near the cutoff values of the tumor thickness categories, where small differences may result in misclassification. Nevertheless, the classification error in only four cSCCs underscores the overall robustness and reliability of EVCM, indicating that the relatively wide range of limits of agreement arises from the small sample size and thus the greater influence of individual outliers.

Furthermore, intraoperative determination of tumor thickness can provide an initial indication of whether a cSCC should be classified as a low-risk (≤6 mm) or high-risk (>6 mm) tumor according to current German guidelines [20]. Although additional histopathological features (e.g., perineural invasion, localization in high-risk areas) are required for a complete decision, the immediate determination of tumor thickness provides valuable prognostic information that can quickly guide further management. This enables early and detailed communication of patient risk, facilitates preliminary planning for interdisciplinary tumor board discussions, and supports timely treatment and follow-up strategies. For example, a tumor thickness > 6 mm warrants an ultrasound examination of the lymph nodes and a more intensive follow-up schedule (every 3–6 months during the first three years), whereas thin tumors (<6 mm) without additional risk factors only require clinical follow-up every 6–12 months for at least three years [20]. Since most cSCCs measured ≤ 6 mm in our collective and there was only one case with a tumor thickness > 6 mm, the validity with regard to high-risk tumors is limited. Further studies with a larger number of cSCCs > 6 mm are necessary to comprehensively evaluate the potential of EVCM in this context. Nevertheless, our study shows that EVCM accurately identified tumors ≤ 6 mm, which represent the majority of cSCCs in the population, as shown by Brantsch et al., who reported that 88% of cSCCs had a tumor thickness of 6 mm or less [10]. Importantly, of the four cSCCs assigned to a higher risk category by EVCM, three were still correctly classified as low-risk (≤6 mm), meaning that only one case potentially resulted in overtreatment due to tumor thickness measurement.

Data presented provide valuable initial findings that form the basis for future research works to confirm our hypothesis in a setting with more patients. In some cases, the EVCM technique, which is based on reflection and fluorescence, made it difficult to clearly distinguish overlying lymphocytes from tumor cells, particularly when the tumor was highly inflamed. This challenge was also observed in moderately differentiated tumors, where the precise identification of the most superficial atypical keratinocytes occasionally proved impaired. While in conventional histopathology, specific pan-cytokeratin antibodies can be used to facilitate this differentiation in very challenging cases [29], rapid staining with such immunohistochemical markers is not yet available for EVCM. In addition, formalin fixation following EVCM imaging cannot be ruled out as a cause of slight tissue shrinkage, potentially leading to minimal overestimation of tumor thickness in this study. There were also some technical challenges. For imaging using EVCM, the fresh tissue must be laid as flat as possible in order to avoid image distortion. Therefore, sectional artifacts and insufficient visualization of the epidermis made it difficult to accurately determine the stratum granulosum when measuring tumor thickness in a small number of cases. Furthermore, the implementation of EVCM is associated with relevant upfront investment costs, primarily related to the acquisition of the imaging platform and the establishment of appropriate digital infrastructure. In addition, personnel training and integration into existing surgical and pathology workflows represent non-negligible implementation efforts. However, these initial expenditures should be weighed against the potential downstream efficiencies enabled by EVCM.

In the future, further studies should be conducted to investigate the intraoperative use of EVCM to measure the tumor thickness of cSCCs in everyday clinical practice. In addition, the use of artificial intelligence could enable the automatic determination of tumor thickness based on digital H&E images by EVCM. This could support further acceleration of hospital processes. In addition, future investigations could research the potential of the EVCM method to reliably identify other high-risk tumor features. Ultimately, this could lead to the initiation of an interdisciplinary tumor board discussion and adjustment of further clinical management immediately after surgery.

## 5. Conclusions

Considering these results, our study demonstrates that the EVCM measurement method is a highly accurate and precise technique for assessing and categorizing tumor thickness, exhibiting strong agreement with the histopathological gold standard and underscoring its potential as an immediate available tool for clinical tumor thickness evaluation. Future studies investigating EVCM’s ability to detect additional high-risk histopathological factors could transform clinical practice by enabling intraoperative precision and revolutionizing the treatment of cSCCs through accelerated therapy decisions and superior patient outcomes.

## Figures and Tables

**Table 1:** Overview of clinical and histopathological characteristics, including tumor localization, thickness, histopathological grade, and measured HTT/CTT values.

Patient Characteristics	
Patients (*n*)	82
Age, mean (years)	78.7
Age, range (years)	60–97
Male patients (%)	76.8
Female patients (%)	23.2
Tumor characteristics	
*Tumor localization*	
Lips (*n*)	2
Ears (*n*)	10
Head and neck (excluding lips and ears) (*n*)	40
Extremities (*n*)	19
Trunk (*n*)	11
*Tumor thickness*	
0–2 mm (*n*)	34
2.1–6 mm (*n*)	47
>6 mm (*n*)	1
*Histopathological grade (degree of differentiation)*	
Well differentiated (G1) (%)	89
Moderately differentiated (G2) (%)	10
Poorly differentiated (G3) (%)	1
Measurements	
HTT, mean (mm)	2.89
HTT, standard deviation (mm)	1.52
HTT, coefficient of variation (%)	53.63
CTT, mean (mm)	2.96
CTT, standard deviation (mm)	1.48
CTT, coefficient of variation (%)	50.05

## Data Availability

The data presented in this study are available on request from the corresponding author. The data are not publicly available due to ethnical and privacy restrictions.

## Abbreviations

The following abbreviations are used in this manuscript:

EVCM	Ex vivo confocal laser scanning microscopy
NMSC	Non-melanocytic skin cancer
cSCC	Cutaneous squamous cell carcinoma
BCC	Basal cell carcinoma
CTT	Confocal tumor thickness
HTT	Histopathological tumor thickness
H&E	Hematoxylin and Eosin
AO	Acridine Orange
IQR	Interquartile range
